# Establishment and Validation of Serum Ferritin Reference Intervals Based on Real-World Big Data and Multi-Strategy Partitioning Algorithms

**DOI:** 10.3390/jcm15030976

**Published:** 2026-01-26

**Authors:** Yixin Xu, Xiaojuan Wu, Junlong Zhang, Qian Niu, Bei Cai, Qiang Miao

**Affiliations:** 1 Department of Laboratory Medicine, West China Hospital, Sichuan University, Chengdu 610041, China; 2 Sichuan Clinical Research Center for Laboratory Medicine, Chengdu 610041, China; 3 Clinical Laboratory Medicine Research Center, West China Hospital, Chengdu 610041, China

**Keywords:** serum ferritin, reference intervals, indirect method, decision tree, Harris–Boyd method

## Abstract

**Background/Objectives:** We aimed to establish and validate population-based reference intervals (RIs) for serum ferritin (SF) using an indirect, date-driven approach based on real-world laboratory data and to optimize partitioning strategies. **Methods:** SF results from 29,723 apparently healthy individuals who underwent health examinations at West China Hospital between 2020 and 2024 were retrospectively analyzed. SF was measured on a Roche Cobas e801 electrochemiluminescence immunoassay platform. After Box–Cox transformation, outliers were removed using an iterative Tukey method. Potential partitioning factors were evaluated, and data-driven age cut-points were explored using decision tree regression and verified with the Harris–Boyd criteria. RIs were estimated using nonparametric percentile methods and validated in an independent cohort of 2494 individuals. **Results:** SF concentrations were significantly higher in males than in females (*p* < 0.001). In females, SF showed a significant positive association with age (*r* = 0.466, *p* < 0.001), whereas no such association was observed in males. Decision tree analysis identified 50 years as the optimal age cut-off for females (R^2^ = 0.2467). The final study-derived RIs were 98.02–997.78 µg/L for males, 10.30–299.55 µg/L for females ≤ 50 years, and 36.61–507.00 µg/L for females > 50 years. In the validation cohort, the study-derived RIs achieved pass rates of 93.83–94.72%, which were significantly higher than the manufacturer-provided RIs (37.12–73.97%, all *p* < 0.001). **Conclusions:** Using a large health examination database and a multi-step partitioning strategy, we established robust sex- and age-specific SF RIs on the Roche Cobas e801 platform for the local population. This work provides a reproducible, generalizable framework for indirect RI determination of other biomarkers.

## 1. Introduction

Serum ferritin (SF) is the primary intracellular iron storage protein and is widely distributed in the liver, spleen, and bone marrow. In clinical practice, SF is a sensitive indicator of body iron stores: low concentrations suggest iron deficiency or iron deficiency anemia, whereas elevated concentrations may reflect iron overload, inflammation, or malignancy. Consequently, SF is frequently used for the diagnosis and monitoring of iron-metabolism disorders [1,2]. However, reference intervals (RIs) for SF—defined here as population-based intervals derived from a reference cohort, which differs from individualized or custom RIs based on an individual’s longitudinal results—vary markedly across populations, regions, and assay platforms [3]. In China, many laboratories still rely on manufacturer-provided RIs derived from foreign populations, which may not account for differences in genetics, diet, and environmental factors [4,5]. Using inadequately established or nontransferable RIs can lead to both false-positive and false-negative interpretations. For SF, an overly narrow upper limit (UL) may label physiological elevations (e.g., in postmenopausal women) as “abnormal”, triggering unnecessary repeat testing, imaging, or referrals, whereas overly broad limits may delay recognition of iron deficiency or overload. In addition, ferritin is an acute-phase reactant, and inappropriate RIs may amplify the confounding effects of subclinical inflammation, metabolic conditions, or liver disease on result interpretation, ultimately undermining clinical decision-making and resource utilization.

Sex and age are well-documented determinants of SF concentrations. Several studies report that females have lower SF concentrations than males, with a pronounced increase after menopause, suggesting that age plays a crucial role in female SF dynamics [6,7]. The Clinical and Laboratory Standards Institute (CLSI) EP28-A3c guideline emphasizes considering partitioning factors, such as sex and age, when establishing Ris [8,9]. However, the RIs currently used in our laboratory (provided by Roche Diagnostics) were derived from a limited cohort of 224 German adults (120 males aged 20–60 years and 104 premenopausal females aged 17–60 years) and lack comprehensive age stratification (males: 30–400 µg/L; females: 13–150 µg/L). Such RIs may underestimate physiological variability, particularly in peri- and postmenopausal women [10,11].

In recent years, indirect methods that utilize large-scale laboratory databases have gained wide acceptance for establishing Ris [12,13]. These approaches reduce subjective bias inherent to direct sampling and have been successfully applied to various clinical biomarkers [14,15,16,17]. In this study, we employed an indirect, date-driven approach using a large real-world laboratory dataset. Decision tree analysis and the Harris–Boyd method were combined to evaluate the necessity of sex- and age-specific partitioning. Our objective was to establish robust, locally applicable RIs for SF to improve diagnostic accuracy and to provide a methodological framework for other biomarkers.

## 2. Materials and Methods

### 2.1. Study Population

We retrospectively collected SF results from 46,963 individuals who underwent routine health examinations at the Health Management Center of West China Hospital, Sichuan University, between January 2020 and December 2024. When an individual had multiple eligible SF measurements during the study period, only the first measurement was retained to ensure independence of observations and to avoid over-representation of frequently tested individuals in the indirect RI estimation. After excluding 702 individuals with incomplete data, 46,261 individuals remained as the initial study population. To ensure biological validity and representativeness of the reference population, we further excluded individuals with abnormal liver function tests (alanine aminotransferase, aspartate aminotransferase), abnormal renal function (serum creatinine), or abnormal routine hematology parameters (hemoglobin, red blood cell count, white blood cell count, platelet count). Subjects with a history of malignancy, recent surgery or hospitalization, active infections, or samples affected by hemolysis, icterus, or lipemia were also excluded. Ultimately, 29,723 apparently healthy individuals (17,846 males and 11,877 females) were included in the RI establishment cohort. An independent validation cohort of 2494 individuals (1490 males, 1004 females) examined between January and May 2025 was used to validate the newly established RIs. This study was approved by the Ethics Committee of West China Hospital, Sichuan University (Approval No. 2022-1682) and was conducted in accordance with the Declaration of Helsinki and relevant institutional guidelines. The requirement for informed consent was waived because only de-identified retrospective data were used.

### 2.2. Instruments and Reagents

SF was measured on a Roche Cobas e801 electrochemiluminescence immunoassay analyzer (Roche Diagnostics GmbH, Mannheim, Germany), using Roche Elecsys^®^ Ferritin reagent kits (Roche Diagnostics GmbH, Mannheim, Germany). Calibration was performed with Roche Elecsys^®^ Ferritin CalSet calibrators, and internal quality control was performed daily using two levels of Roche Elecsys^®^ PreciControl Tumor Marker controls (low and high). Westgard multi-rules (1_3S_, 2_2S_, R_4S_) were applied. The laboratory’s analytical performance specification for SF during the study period was CV ≤ 5% (per the immunoassay quality management plan), and the cumulative CVs for both QC levels remained within this target. The laboratory participated continuously in external quality assessment/proficiency testing organized by the National Center for Clinical Laboratories (NCCL, Beijing, China) and the College of American Pathologists (CAP), consistently achieving satisfactory performance throughout the study period.

### 2.3. Establishment of Reference Intervals

Initially, data normality was assessed visually using histograms. Data exhibiting evident skewness underwent a Box–Cox transformation in R software (version 3.6.3) to approximate a normal distribution. The optimal transformation parameter (λ) was determined by maximum likelihood estimation (Formula (1)). Histograms were then used to re-evaluate the distribution of the transformed data.

(1) $$Y(\lambda )=\left\{\begin{array}{c} \frac{X^{\lambda }-1}{\lambda }\;\;\;\lambda \neq 0\\ ln(X)\;\;\;\;\;\lambda =0 \end{array}\right\}$$

**Figure 1:**
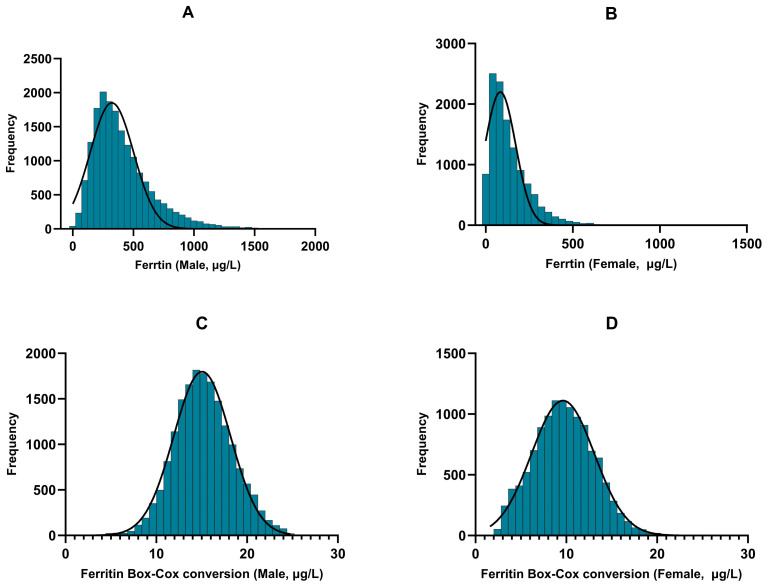
Normal distribution diagram of serum ferritin in male and female before and after Box–Cox transformation (λ = 0.284). (**A**) Histogram of serum ferritin concentrations in males before transformation. (**B**) Histogram of serum ferritin concentrations in females before transformation. (**C**) Histogram of Box–Cox transformed serum ferritin values in males. (**D**) Histogram of Box–Cox transformed serum ferritin values in females. Solid lines represent the fitted normal density curves.

In Formula (1), X represents the original data and λ is the transformation parameter chosen to best approximation a normal distribution. For positive X values, the Box–Cox transformation takes various forms: λ = 2 corresponds to a square transformation, λ = 0.5 to a square root transformation, and λ = 0 to a natural logarithmic transformation. In practice, statistical software such as R (version 3.6.3) or Python (version 3.13.1) is used to determine the optimal value of λ that maximizes the likelihood of normality. In this study, λ was set to 0.284 and the Box–Cox transformation successfully converted the distribution of SF data from non-normal to approximately normal, with details provided in Table 1 and Figure 1.

The Tukey method was applied iteratively to identify and remove outliers, defined as values below P_25_ − 1.5 × IQR or above P_75_ + 1.5 × IQR until no further outliers remained [18]. After data normalization and cleaning, RI partitioning was evaluated in two stages. First, sex-specific differences were assessed using the Harris–Boyd standard normal deviate method (Formula (2)). Second, for age (continuous variable), scatterplots were inspected and decision tree regression was implemented to explore data-driven age cut-points for RI partitioning. Age was modeled as a continuous predictor and SF (Box–Cox transformed) as the response. A recursive partitioning algorithm was fitted to minimize within-node variance (equivalently, maximize between-node separation) and to propose split points that improved model fit (R^2^). R^2^ is the measure of fitting degree for all subclasses after every division step in each stage, ranging from 0 (no fit) to 1 (exact fit). The age partition point corresponding to the highest R^2^ was selected as the optimal threshold [19,20]. Candidate age partitions were subsequently confirmed by the Harris–Boyd criteria (*Z* and Z*); partitioning was considered statistically justified When *Z* > Z* and the standard deviation ratio (SD ratio) exceeded 1 [9].

(2) $$\begin{array}{c} Z=\frac{\left|{\bar{X}}_{1}-{\bar{X}}_{2}\right|}{{\left[\left(\frac{{S_{1}}^{2}}{N_{1}}\right)+\left(\frac{{S_{2}}^{2}}{N_{2}}\right)\right]}^{\frac{1}{2}}}\\ Z^{*}=3{\left[\frac{\left(N_{1}+N_{2}\right)}{240}\right]}^{\frac{1}{2}}(N_{1}\geq 120,N_{2}\geq 120) \end{array}$$

In Formula (2), ${\bar{X}}_{1}$ and ${\bar{X}}_{2}$ are the means, $S_{1}$ and $S_{2}$ are the standard deviations, and $N_{1}$ and $N_{2}$ are the sample sizes of the two groups being compared.

Finally, RIs were estimated using nonparametric methods (2.5th–97.5th percentiles) and 90% confidence interval (CI) were computed by bootstrap resampling. To assess clinical applicability, we evaluated the proportion of individuals in the validation cohort whose SF values fell outside these intervals in accordance with CLSI EP28-A3c guidelines. An RI was considered valid if fewer than 10% of individuals had values outside the interval.

### 2.4. Statistical Analysis

Statistical analyses were performed using SPSS v23.0 (IBM Corp., Armonk, NY, USA) and R (version 3.6.3, 2020). R was used for Box–Cox transformation, iterative Tukey outlier exclusion, decision tree regression, Harris–Boyd calculations (Z, Z* and SD ratio), non-parametric percentile RI estimation, and graphical visualization. SPSS was used for descriptive statistics and hypothesis testing. Data normality was evaluated using histograms and skewness-kurtosis tests. Normally distributed variables were expressed as mean ± standard deviation and analyzed by independent samples *t*-tests. Categorical data were expressed as counts (percentages) and analyzed using chi-squared tests. Pearson correlation and simple linear regression were used to assess associations be-tween SF and age. Statistical significance was set at *p* < 0.05.

## 3. Results

### 3.1. SF Data Characteristics

A total of 29,723 apparently healthy individuals (17,846 males and 11,877 females) were included in the final analysis. Initial assessment revealed a markedly positive skewed in the SF distribution for both sexes (Figure 1A,B). Following Box–Cox transformation, the distributions approximated normality, with marked improvements in skewness and kurtosis (Figure 1C,D; Table 1). Specifically, in males, skewness decreased from 1.681 to 0.190 and kurtosis from 4.512 to 0.350. In females, skewness decreased from 1.952 to 0.163 and kurtosis decreased from 6.478 to −0.254. After iterative outlier removal using the Tukey method, the final dataset for RI modeling comprised 17,568 males (aged 16–91 years, mean SF 403.72 ± 230.03 µg/L) and 11,831 females (aged 14–91 years, mean SF 133.93 ± 108.89 µg/L) (Table 1).

### 3.2. RI Partitioning Analysis

**Figure 2:**
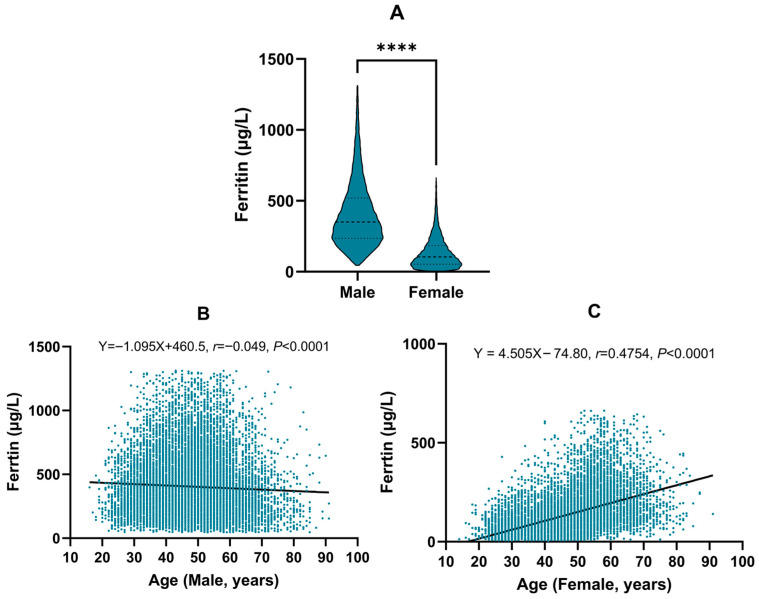
Evaluation of sex and age as partitioning factors for SF. (**A**) Comparison of SF concentrations between males and females. (**B**) Correlation analysis between SF concentrations and age in males. (**C**) Correlation analysis between SF concentrations and age in females. **** indicates *p* < 0.0001.

To determine whether sex- and age-specific partitioning of SF RIs was necessary, we compared SF concentrations between males and females after outlier exclusion. Between-sex differences were substantial (*p* < 0.001; Figure 2A) and met the Harris–Boyd criteria for partitioning (*Z* > Z* and SD ratio = 2.11; Table 2). We next evaluated age as a partitioning factor within each sex. In males, SF showed only a weak inverse correlation with age (*r* = −0.049, *p* < 0.001; Figure 2B) and decision tree regression suggested a split at 61 years with poor model fit (R^2^ = 0.0102; Table 3); this split was not supported by the Harris–Boyd test (*Z* < Z*; Table 2). In females, SF increased markedly with age (*r* = 0.466, *p* < 0.001; Figure 2C) and the decision tree regression identified 50 years as the optimal cut-off with a substantially better model fit (R^2^ = 0.2467, Table 3), which was subsequently confirmed by the Harris–Boyd method (*Z* > Z* and SD ratio = 1.55; Table 2). Therefore, the final partitioning scheme consisted of a single group for males and two age-based subgroups for females (≤50 years and >50 years).

### 3.3. Establishment of SF Reference Intervals

Based on the final partitioning scheme, the 95% distribution RIs for SF in this regional healthy population were established using the nonparametric percentile method (*P*_2.5_–*P*_97.5_). The resulting RIs were 98.02–997.78 µg/L for males aged 16–91 years; 10.30–299.55 µg/L for females aged 14–50 years; and 36.61–507.00 µg/L for females aged > 50 years (Table 4). For comparison, the manufacturer-provided RIs are 30.00–400.00 µg/L for males and 13.00–150.00 µg/L for females without age stratification. Notably, the ULs of the study-derived RIs exceeded the manufacturer’s ULs, particularly in females > 50 years (Table 5).

### 3.4. Validation and Comparison of RIs

To evaluate the clinical applicability of the study-derived RIs, validation was conducted using an independent cohort of 2494 individuals. The pass rates of the study-derived RIs were significantly higher than those of the manufacturer-provided RIs across all subgroups: 93.83% vs. 56.71% in males (*p* < 0.001); 94.72% vs. 73.97% in females aged ≤ 50 years (*p* < 0.001); and 94.52% vs. 37.12% in females aged > 50 years (*p* < 0.001).

## 4. Discussion

Population-based RIs are fundamental for interpreting laboratory results and for minimizing misclassification in clinical decision making. SF, as the primary form of iron storage in the body, plays an important clinical role in assessing iron metabolism, aiding in the diagnosis of iron-deficiency anemia, iron overload, and certain malignancies [2,21,22]. However, SF concentrations are influenced by multiple factors including sex, age, ethnicity, geographic region, and analytical platform, resulting in considerable variability in RIs across populations [3,5,7,16]. Therefore, establishing locally appropriate RIs based on large regional datasets is essential to ensure accurate interpretation of laboratory results [13].

In this study, by using a large real-world health examination dataset and an indirect RI approach, we established and validated sex- and age-specific SF RIs for apparently healthy individuals in southwestern China. We observed that SF concentrations were significantly higher in males than in females, consistent with previous reports [23,24], reflecting differences in iron storage between sexes that may be attributable to physiological factors, greater muscle mass, and differences in estrogen regulation of iron metabolism [25]. After applying a multi-strategy partitioning algorithm, no further age partitioning was required for males, and the overall male RI was 98.02–997.78 µg/L. For females, our analysis clearly identified 50 years as an important threshold, with SF levels significantly higher in females aged > 50 years compared to those aged ≤ 50 years. The RIs established were 10.30–299.55 µg/L for females aged ≤ 50 years and 36.61–507.00 µg/L for females aged > 50 years. This finding is consistent with results from other studies using different analytical platforms in China [5], and supports the physiological increase in iron storage following menopause due to cessation of menstrual blood loss [7,10]. The observed sex and age patterns are biologically plausible. Compared with women of reproductive age, men typically have higher iron stores because they lack menstrual iron loss and may have greater dietary iron intake and body iron reserves [26]. In women, the rise in SF after midlife is consistent with the reduction and eventual cessation of menstrual blood loss after menopause, together with hormonal regulation of iron metabolism and hepcidin signaling [27,28]. These physiological changes support the need for age-specific interpretation in females and help explain why a single female RI without age stratification may lead to frequent false-positive results in older women.

Notably, the ULs of the study-derived RIs—particularly in females > 50 years—exceeded those provided by the manufacturer. This may reflect both true physiological variability in the local cohort and the right-tailed distribution of ferritin, for which the 97.5th percentile may remain sensitive to residual heterogeneity even after rigorous exclusion criteria and iterative outlier removal. Because SF is also an acute-phase reactant, unrecognized low-grade inflammation and metabolic or hepatic factors (e.g., fatty liver), alcohol intake, or unrecorded supplement use may contribute to higher SF values in an “apparently healthy” dataset. Therefore, SF values near the UL should be interpreted in clinical context rather than in isolation. For clinical implementation, these RIs are intended for adults comparable to the health examination population and measured using the same analytical platform. Importantly, an RI is not a diagnostic decision limit: some patients with disease may still have SF values within the RI, and values outside the RI do not by themselves establish a diagnosis. When SF is above the UL, repeat testing and evaluation with complementary indices (e.g., hemoglobin and red cell indices, transferrin saturation, C-reactive protein, and liver function tests) can help distinguish iron overload from inflammation-related hyperferritinemia; when SF is low or borderline, assessment of iron deficiency should similarly integrate clinical context and additional iron studies [1,2,4]. Where available, interpretation should also consider prior results, longitudinal trends and clinical suspicion.

Methodologically, this study followed CLSI EP28-A3c recommendations, used a transparent transformation and outlier strategy, and combined decision tree analysis with Harris–Boyd testing to provide an objective, reproducible partitioning workflow. Such approaches have been widely used in recent pediatric RI studies [29,30,31], and are becoming an important trend in indirect RI modeling. The RIs established in this study underwent external verification in an independent cohort and yielded pass rates > 93.8% in all subgroups, substantially higher than the manufacturer-provided RIs, supporting improved local applicability. Similar findings have been reported in other studies both in China and internationally [32,33,34], suggesting that manufacturer-provided RIs, typically derived from selected populations in other countries, may not adequately reflect the actual status of local populations, potentially leading to overdiagnosis, unnecessary anxiety, and inefficient use of healthcare resources [35].

Nonetheless, this study has certain limitations including the single-center retrospective design, the lack of individual information on diet, supplementation, alcohol intake and comorbidities, and platform specificity. In addition, while finer age stratification (e.g., by decades) may reveal gradual trends, excessive partitioning can compromise statistical robustness and was not justified by our objective partitioning criteria in this dataset. Future studies incorporating multicenter datasets and different analytical systems are needed to improve the representativeness and broader applicability of the RIs.

## 5. Conclusions

In summary, using a large real-world health examination dataset and an indirect modeling strategy, we established population-based serum ferritin RIs for individuals on the Roche Cobas e801 platform. These RIs offer improved regional representativeness and clinical utility, providing robust support for the accurate diagnosis of iron metabolism disorders. The proposed framework is reproducible and may be extended to other biomarkers using real-world laboratory databases.

## Figures and Tables

**Table 1:** Skewness and kurtosis of serum ferritin distribution before and after Box–Cox transformation, with and without outlier exclusion.

	*N*	Before Transformation				After Transformation			
		Mean	SD	Skewness (95% CI)	Kurtosis (95% CI)	Mean	SD	Skewness (95% CI)	Kurtosis (95% CI)
Outliers undeleted									
Male	17,846	413.55	258.92	1.681 (1.596, 1.766)	4.512 (3.942, 5.056)	15.27	3.27	0.190 (0.148, 0.235)	0.350 (0.263, 0.438)
Female	11,877	136.50	116.50	1.952 (1.774, 2.147)	6.478 (4.651, 8.590)	9.73	3.30	0.163 (0.124, 0.197)	−0.254 (−0.337, −0.166)
Outliers deleted									
Male	17,568	403.72	230.03	1.130 (1.095, 1.166)	1.190 (1.068, 1.323)	15.22	3.06	0.138 (0.112, 0.165)	−0.273 (−0.315, −0.233)
Female	11,831	133.93	108.89	1.482 (1.425, 1.539)	2.556 (2.278, 2.820)	9.69	3.24	0.085 (0.054, 0.115)	−0.447 (−0.492, −0.399)

Abbreviations: SD, standard deviation; CI, Confidence interval. Note: Mean and SD before transformation are in µg/L; statistics after transformation are reported on the transformed scale for distributional assessment.

**Table 2:** Standard Normal Deviate Test Results for Sex and Age Partitions in RI Estimation.

Gender/Age	*N*	Mean + SD	*Z*	Z*	SD Ratio ^†^	Partitioning Recommended
Male	17,568	403.72 ± 230.03	134.66	33.20	2.11	Yes
Female	11,831	133.93 ± 108.89				
Male						
≤61	15,872	411.31 ± 230.85	14.55	25.67	1.10	No
>61	1696	332.70 ± 209.25				
Female						
≤50	7469	92.60 ± 76.86	55.86	21.06	1.55	Yes
>50	4362	204.69 ± 118.81				

Abbreviations: RI, reference interval; SD, standard deviation. ^†^: SD ratio was calculated by dividing the larger SD by the smaller SD.

**Table 3:** Optimal Age Split Point Determined by the Decision Tree Method.

	Gender	Best Split Point (Years)	R^2^ *
Ferritin (µg/L)	Male	61	0.0102
	Female	50	0.2467

*: R^2^ is the measure of fitting degree for all subclasses after every division step in each stage, higher values suggest a better fit.

**Table 4:** The study-derived and manufacturer’s reference intervals of serum ferritin.

Gender	Age (Years)	N	Study-Derived RI (µg/L)	90% CI for LL	90% CI for UL	Manufacturer’s RI (µg/L)
Female	14–50	7469	10.30–299.55	9.99–10.70	287.03–307.00	13.00–150.00 *
	51–91	4362	36.61–507.00	32.92–39.31	497.52–521.00	
Male	16–91	17,568	98.02–997.78	96.42–99.92	983.78–1011.00	30.00–400.00

Abbreviations: RI, reference interval; LL, lower limit; UL, upper limit; CI, confidence interval. *: Reference intervals for female were not grouped by age.

**Table 5:** Comparison of verification results between study-derived and manufacturer’s reference intervals for serum ferritin.

Subgroup	N	Study-Derived RI Verification Pass Rate (n)	Manufacturer’s RI Verification Pass Rate (n)	*p*
Female (≤50 years)	511	94.72% (484)	73.97% (378)	<0.001
Female (>50 years)	493	94.52% (466)	37.12% (183)	<0.001
Male (all ages)	1490	93.83% (1398)	56.71% (845)	<0.001

Abbreviations: RI, reference interval.

## Data Availability

The original contributions presented in this study are included in the article. Further inquiries can be directed to the corresponding author.

## Abbreviations

The following abbreviations are used in this manuscript:

CAP	College of American Pathologists
CI	Confidence interval
CLSI	Clinical and Laboratory Standards Institute
CV	Coefficient of variation
IQR	Interquartile range
NCCL	National Center for Clinical Laboratories
QC	Quality control
RI	Reference interval (population-based)
SF	Serum ferritin
SD	Standard deviation
LL	Lower limit
UL	Upper limit

## References

[1] Camaschella C. (2019). Iron deficiency. Blood.

[2] Pasricha S.R., Tye-Din J., Muckenthaler M.U., Swinkels D.W. (2021). Iron deficiency. Lancet.

[3] Truong J., Naveed K., Beriault D., Lightfoot D., Fralick M., Sholzberg M. (2024). The origin of ferritin reference intervals: A systematic review. Lancet Haematol..

[4] Sezgin G., Monagle P., Loh T.P., Ignjatovic V., Hoq M., Pearce C., McLeod A., Westbrook J., Li L., Georgiou A. (2020). Clinical thresholds for diagnosing iron deficiency: Comparison of functional assessment of serum ferritin to population based centiles. Sci. Rep..

[5] Wang Q.P., Guo L.Y., Lu Z.Y., Gu J.W. (2020). Reference intervals established using indirect method for serum ferritin assayed on Abbott Architect i2000(SR) analyzer in Chinese adults. J. Clin. Lab. Anal..

[6] Floegel A., Intemann T., Siani A., Moreno L.A., Molnar D., Veidebaum T., Hadjigeorgiou C., De Henauw S., Hunsberger M., Eiben G. (2024). Cohort-Based Reference Values for Serum Ferritin and Transferrin and Longitudinal Determinants of Iron Status in European Children Aged 3–15 Years. J. Nutr..

[7] Addo O.Y., Mei Z., Jefferds M.E.D., Jenkins M., Flores-Ayala R., Williams A.M., Young M.F., Luo H., Ko Y.-A., Papassotiriou I. (2025). Physiologically based serum ferritin thresholds for iron deficiency among women and children from Africa, Asia, Europe, and central America: A multinational comparative study. Lancet Glob. Health.

[8] Harris E.K., Boyd J.C. (1990). On dividing reference data into subgroups to produce separate reference ranges. Clin. Chem..

[9] CLSI (2010). Defining, Establishing, and Verifying Reference Intervals in the Clinical Laboratory; Approved Guideline.

[10] Ahanchi N.S., Khatami F., Llanaj E., Quezada-Pinedo H.G., Dizdari H., Bano A., Glisic M., Eisenga M.F., Vidal P.-M., Muka T. (2024). The complementary roles of iron and estrogen in menopausal differences in cardiometabolic outcomes. Clin. Nutr..

[11] Cancado R.D., Leite L.A.C., Munoz M. (2025). Defining Global Thresholds for Serum Ferritin: A Challenging Mission in Establishing the Iron Deficiency Diagnosis in This Era of Striving for Health Equity. Diagnostics.

[12] Jones G.R.D., Haeckel R., Loh T.P., Sikaris K., Streichert T., Katayev A., Barth J.H., Ozarda Y., Null N. (2018). Indirect methods for reference interval determination—Review and recommendations. Clin. Chem. Lab. Med..

[13] Doyle K., Bunch D.R. (2023). Reference intervals: Past, present, and future. Crit. Rev. Clin. Lab. Sci..

[14] Miao Q., Lei S., Chen F., Niu Q., Luo H., Cai B. (2024). A preliminary study on the reference intervals of serum tumor marker in apparently healthy elderly population in southwestern China using real-world data. BMC Cancer.

[15] Wei B., Guo Y., Zhang L., Zhong H., Miao Q., Yan L., Bai Y., Feng W., Liu W., Niu Q. (2021). Reference ranges of T lymphocyte subsets by single-platform among healthy population in southwest China. BMC Immunol..

[16] Dirks N.F., den Elzen W.P.J., Hillebrand J.J., Jansen H.I., Boekel E.T., Brinkman J., Buijs M.M., Demir A.Y., Dijkstra I.M., Endenburg S.C. (2024). Should we depend on reference intervals from manufacturer package inserts? Comparing TSH and FT4 reference intervals from four manufacturers with results from modern indirect methods and the direct method. Clin. Chem. Lab. Med..

[17] Jansen H.I., Dirks N.F., Hillebrand J.J., Ten Boekel E., Brinkman J.W., Buijs M.M., Demir A.Y., Dijkstra I.M., Endenburg S.C., Engbers P. (2024). Age-Specific Reference Intervals for Thyroid-Stimulating Hormones and Free Thyroxine to Optimize Diagnosis of Thyroid Disease. Thyroid.

[18] Johansen M.B., Christensen P.A. (2018). A simple transformation independent method for outlier definition. Clin. Chem. Lab. Med..

[19] Putora P.M., Panje C.M., Papachristofilou A., Dal Pra A., Hundsberger T., Plasswilm L. (2014). Objective consensus from decision trees. Radiat. Oncol..

[20] Peng X., Lv Y., Feng G., Peng Y., Li Q., Song W., Ni X. (2018). Algorithm on age partitioning for estimation of reference intervals using clinical laboratory database exemplified with plasma creatinine. Clin. Chem. Lab. Med..

[21] Ramirez-Carmona W., Diaz-Fabregat B., Yuri Yoshigae A., Musa de Aquino A., Scarano W.R., de Souza Castilho A.C., Marsicano J.A., Prado R.L.D., Pessan J.P., Mendes L.d.O. (2022). Are Serum Ferritin Levels a Reliable Cancer Biomarker? A Systematic Review and Meta-Analysis. Nutr. Cancer.

[22] Shesh B.P., Connor J.R. (2023). A novel view of ferritin in cancer. Biochim. Biophys. Acta Rev. Cancer.

[23] Li S., Lin L., Mo Z., Qin X., Lv H., Gao Y., Tan A., Yang X., Huang S., Chen Z. (2011). Reference values for serum ferritin in Chinese Han ethnic males: Results from a Chinese male population survey. Clin. Biochem..

[24] Han L.L., Wang Y.X., Li J., Zhang X.L., Bian C., Wang H., Du S., Suo L. (2014). Gender differences in associations of serum ferritin and diabetes, metabolic syndrome, and obesity in the China Health and Nutrition Survey. Mol. Nutr. Food Res..

[25] Rushton D.H., Barth J.H. (2010). What is the evidence for gender differences in ferritin and haemoglobin?. Crit. Rev. Oncol. Hematol..

[26] Merlo F., Groothof D., Khatami F., Ahanchi N.S., Wehrli F., Bakker S.J.L., Eisenga M.F., Muka T. (2023). Changes in Iron Status Biomarkers with Advancing Age According to Sex and Menopause: A Population-Based Study. J. Clin. Med..

[27] Kastrati L., Groothof D., Quezada-Pinedo H.G., Raeisi-Dehkordi H., Bally L., De Borst M.H., Bakker S.J., Vidal P.-M., Eisenga M.F., Muka T. (2024). Utility of iron biomarkers in differentiating menopausal status: Findings from CoLaus and PREVEND. Maturitas.

[28] Kim M., Chang Y., Cho Y., Kwon M.J., Joh H.K., Lim G.Y., Kwon R., Ahn J., Park J., Kim K.-H. (2025). Accelerated increase in ferritin levels during menopausal transition as a marker of metabolic health. Sci. Rep..

[29] Yan R., Li K., Lv Y., Peng Y., Van Halm-Lutterodt N., Song W., Peng X., Ni X. (2022). Comparison of reference distributions acquired by direct and indirect sampling techniques: Exemplified with the Pediatric Reference Interval in China (PRINCE) study. BMC Med. Res. Methodol..

[30] Peng X., Peng Y., Zhang C., Zhao M., Yang H., Cao S., Li G., Jiang Y., Guo Z., Chen D. (2022). Reference intervals of 14 biochemical markers for children and adolescence in China: The PRINCE study. Clin. Chem. Lab. Med..

[31] Song W., Yan R., Peng M., Jiang H., Li G., Cao S., Jiang Y., Guo Z., Chen D., Yang H. (2022). Age and sex specific reference intervals of 13 hematological analytes in Chinese children and adolescents aged from 28 days up to 20 years: The PRINCE study. Clin. Chem. Lab. Med..

[32] Snozek C.L.H., Spears G.M., Porco A.B., Erb S., Kaleta E.J., Bryant S.C., Baumann N.A. (2021). Updated ferritin reference intervals for the Roche Elecsys(R) immunoassay. Clin. Biochem..

[33] Wang Y., Yu X., Fan J., Huang M., Fu Y., Zhang H., Kong R., Man Q. (2023). Serum Ferritin in Geriatric Individuals in the Shanghai Region: Distribution, Correlations, and Reference Intervals. Clin. Lab..

[34] Rodgers S., Woolley T., Smith J., Prinsloo P., Fernando N. (2024). Updated adult ferritin reference intervals based on a large, healthy UK sample, measured on Roche Cobas series analysers. Ann. Clin. Biochem..

[35] Kurstjens S., van Dam A.D., Oortwijn E., den Elzen W.P.J., Candido F., Kusters R., Schipper A., Kortmann Y.F., Herings R.M., Kok M. (2025). Inconsistency in ferritin reference intervals across laboratories: A major concern for clinical decision making. Clin. Chem. Lab. Med..

